# Ethnobotanical study of nutraceutical plants used to manage opportunistic infections associated with HIV/AIDS in Acholi sub-region, Northern Uganda

**DOI:** 10.1186/s41182-023-00540-w

**Published:** 2023-09-01

**Authors:** Norah Ikinyom, Alice Veronica Lamwaka, Aloysius Tenywa Malagala, Elly Kurobuza Ndyomugyenyi

**Affiliations:** 1 Department of Environment, Faculty of Agriculture and Environment, Gulu University, P.O. Box 166, Gulu, Uganda; 2 Department of Pharmacology, Faculty of Medicine, Gulu University, P.O. Box 166, Gulu, Uganda; 3 Institute of Peace and Strategic Studies, Gulu University, P.O. Box 166, Gulu, Uganda; 4 Department of Animal Production and Range Management, Faculty of Agriculture and Environment, Gulu University, P.O.Box 166, Gulu, Uganda

**Keywords:** Ethnobotany, HIV/AIDS, Nutraceutical plants, Opportunistic infections, Safety

## Abstract

**Background:**

Nutraceutical plants play a potential role as supportive treatment with antiretroviral drugs in the management of opportunistic infections associated with HIV/AIDS. There is limited documentation of nutraceutical plants in Northern Uganda and limited literature addressing processes to be adopted for quality assurance of herbal formulations in Uganda. This study aimed to document plant species with nutritional and medicinal properties used for managing opportunistic infections associated with HIV/AIDS by traditional medicine practitioners (TMPs), who included professional herbalists, herbal farmers and herbal sellers in Acholi sub region, Northern Uganda.

**Methods:**

An ethnobotanical study was carried out in Amuru, Gulu and Pader districts, Northern Uganda. Cross-sectional data were collected using purposive and snowball sampling techniques. A total of four hundred (378 women and 22 men) informants were selected. Data were collected using semistructured interviews, focus group discussions and direct observation. Data were analysed using descriptive statistics, familiarity index (FI), fidelity level (FL) and informant consensus factor (ICF).

**Results:**

This study recorded a total of 84 nutraceutical plant species, which are used to manage opportunistic infections associated with HIV/AIDS. Most abundant families were Leguminoceae, Asteraceae and Solanaceae. Six nutraceutical plants that had higher fidelity level and familiarity index values included *Momordica foetida* Schumach, *Erigeron floribundus* (Kunth) Sch. Bip, *Mangifera indica* L, *Cajanus cajan* L. Millsp, *Eucalyptus globulus* Labill and *Cucurbita pepo* L. Respondents’ knowledge on nutraceutical plants had a positive significant correlation with age (*R*^2^ = 0.0524, *p* ≤ 0.01). The popular mode of preparation are decoctions/boiling in water (70%), while the most used route of administration is oral (76%). TMPs tried to ensure that during collection and processing, plant materials were free from contamination. TMPs reported limited knowledge on preservation techniques.

**Conclusions:**

This study demonstrates the availability and diversity of nutraceutical plants in Uganda and reports methods of processing and administration used by TMPs. Both men and women used nutraceutical plants to manage opportunistic infections associated with HIV/AIDS and showed great extent of their traditional knowledge. Most of the nutraceutical plants in study area are wild and abundant; however, high percentage use of plant roots and bark threatens the sustainable use from the wild.

## Introduction

Globally, 38.4 million people were living with human immunodeficiency virus/acquired immune deficiency syndrome (HIV/AIDS) in 2021 [1]. Of these, 25.6 million people were in Eastern and Southern Africa alone [1]. Currently, the number of people with HIV in Uganda is estimated to be about 1.4 million [2] and the national prevalence rate of HIV/AIDS is 6.4%, Gulu district is at 13%, Amuru district at 4.5% and Pader district at 8.5% [2–4]**.**

A weak immune system in patients with HIV/AIDS infection results in patients being susceptible to opportunistic infections, such as oral candidiasis and genital infections that are caused by opportunistic pathogens, such as *Candida albicans* [2–4]. Other opportunistic pathogens include *Staphylococcus aureus*, *Mycobacterium tuberculosis*, *Streptococcus pneumoniae*, *Klebsiella pneumoniae*, *Cryptococcus neoformans* and *Pseudomonas aeruginosa* [5, 6]. There are no vaccines for HIV, but people living with HIV/AIDS, who have a timely diagnosis and access to medication, proper care and treatment with effective antiretroviral drugs can lead normal, healthy and productive lives and are expected to live nearly as long as the general population [7–9]. However, in resource poor countries, such as Uganda, the use of antiretroviral (ARV) drugs in managing opportunistic infections by HIV positive patients is limited by toxic side effects, poor adherence to treatment and limited access to the drugs [10, 11]. Antimicrobial resistance (AMR) due to continued use of antibiotics to which pathogens such as bacteria and fungi have developed resistance, has been reported [10, 12, 13]. The increasing prevalence of HIV-1 drug resistance in low and middle income countries hinders successful ART [14]. Even when several pharmaceutical drugs are available at subsidized cost, most Ugandans cannot afford to buy modern medicine due to their low income status [15]. Other factors, such as high exposure to infectious agents, poverty and malnutrition, have all led to significant crisis in the management of HIV/AIDS [10, 11]. Opportunistic infections due to HIV results in morbidity and mortality and this loss of labour force results in loss of productivity, profitability and hence poverty [16, 17]. There is a high need to explore and develop reliable, safe drugs and supportive treatment from natural resources to manage HIV infection [18].

One of the potential ways of managing opportunistic infections associated with HIV/AIDS could be nutraceutical plants, as sources of new antimicrobial molecules [12]. The research and development of alternative safe drugs should follow standards, parameters and protocols to ensure quality control is guaranteed [19, 20]. Nutraceutical plants are the most popular form of traditional medicine, and are used worldwide as an alternative and/or complementary medicine [21–23]. Nutraceutical plants are plants with nutritional or medicinal properties or both [24]. Medicinal plants are plants that exert beneficial pharmacological effect on the human or animal body or possess therapeutic properties [24]. Living organisms can grow, maintain themselves and reproduce by assimilation of nutritious plants that contain vitamins A, C, K, Fibre, Riboflavin and minerals, which are essential requirements for the health of HIV positive patients [25]. Nutraceutical plants have a potential in the management of opportunistic infections associated with of HIV/AIDS especially among rural poor [18].

There have been reports of nutraceutical plant use worldwide, such as in Pakistan, *Cassia fistula* and *Punica granatum* have been used against fungal opportunistic infections associated with HIV [26]. Approximately, 137 medicinal plants were used to treat pneumonia and tuberculosis opportunistic infections in Pakistan [27]. In Thailand, 12 medicinal plants used among HIV patients, were evaluated for antibacterial activities [28].

Ethnobotanical studies conducted in different African countries [29–36] reported that nutraceutical plants were being used to manage a wide range of diseases. For example, ninety four (94) plant species were reported to be used in managing HIV/AIDS opportunistic infections in Livingstone, Zambia [33]. Seventeen (17) plants located in Western and Eastern Cape, South Africa were reported as being used in the management of HIV and related opportunistic infections [37]. Thirty three (33) plant species were identified in the management of opportunistic fungal infections (O.F.I) by HIV/AIDS patients in the Amathole district of the Eastern Cape Province, South Africa [34]. Nagata et al. [38] identified 40 medicinal plant species used among people living with HIV/AIDS in Suba district, Kenya.

Poor health facilities and limited access to antiretroviral drugs in Uganda have perpetuated and increased the use of nutraceutical plants, especially in rural areas for opportunistic ailments of HIV/AIDS [39]. Nutraceutical plant, *Albizia coriaria* Oliv. is widely used in traditional medicine for treating opportunistic infections, such as skin infections, cough, syphilis, and sore throat among PLHIV in Uganda [12, 40, 41]. The root of *Serudaca pedunculata* Fresen. is commonly used in Uganda for treating STDs, TB, Coughs [12]. Ethnobotanical survey by Shehu et al. [32] in Buikwe district, Uganda indicated that 52 plants were used in the management of HIV/AIDS opportunistic infections.

There is limited documentation of nutraceutical plants in Northern Uganda and limited literature addressing processes to be adopted for quality assurance of herbal formulations in Uganda. This ethnobotanical study aimed to document plant species with nutritional and medicinal potentials used for managing opportunistic infections associated with HIV/AIDS by traditional medicine practitioners (TMPs) in Acholi sub region, Northern Uganda. This study reported the quality control methods employed by TMPs and the prioritized problems encountered during harvest, processing, production and administration of the documented nutraceutical plants. This study also provided information on the local solutions to prioritized problems and recommendations to the government on opportunities to help with quality control measures.

## Methods

### Description of the study area

Uganda is a landlocked country that lies astride the equator and covers an area of 236,000 square kilometres comprising dryland, open water and permanent swamp [42]. Uganda population census of 2013 [43], reported approximately 7.3 million households with a population of 34.1 million were in Uganda [43]. There are 56 tribes and 9 indigenous communities [43]. There are approximately 5000 species of higher plants in Uganda, of which 70 are endemic [42]. There are more than 200 species of non-cultivated edible plants and 75 species of edible fruit trees in Uganda, while forestland covers approximately 3.3 million hectares [42]. Ethnobotanical research in Uganda has identified more than 300 trees, shrubs and herbs growing wild associated with medicinal value [42]. Traditional knowledge of plants with medicinal value is passed on from one generation to another [42].

The agricultural sector is composed of crop and animal production, forestry, fisheries and major crops produced include cotton, maize, tea, sugarcane, bananas [42]. According to livestock census, Uganda had fourteen point two (14.2) million cattle, 16 million goats, 6 million sheep, 47.6 million poultry and 4.1 million pigs [42]. This study was carried out in Amuru, Gulu and Pader districts. In general, altitude ranges between 1000 and 1200 Metres above sea level [42]. The districts experience tropical climate with average annual rainfall of 1507 mm and average temperatures is 23 °C, vegetation is intermediate savannah grassland [42]. The districts have established health centres. Despite the presence of established health centres, people are still reported to seek medical attention from traditional medicine practitioners [44, 45]

Gulu district is located at 2° 46′ 54.0″ N and 32° 17′ 57.0″ E in Northern Uganda [42]. Main economic activity of ninety percent (90%) of the population is subsistence agriculture [43]. The climate is tropical wet and dry, according to Koppen–Geiger climate classification system [46]. Total population in Gulu district is 275,613, while total households are 55,441 [43]

Pader district, homeland of Acholi ethnic group is located in Northern Uganda at 2° 49′ 59.99″ N and 33° 04′ 60.00″ E [42]. Total area is 3.362 square kilometres. Food crops grown are beans, peas, cassava, cotton, groundnuts, and sunflower. Ninety percent (90%) of the economic activity is subsistence agriculture. The total population in Pader district is 178,000, while total households are 34,183 [43]

Amuru district located at 2° 46′ 54″ N and 32° 17′ 57″ E in Northern Uganda. Ninety-eight percent (98%) of the economic activity is subsistence agriculture [42]. Total area is 3625.9 square kilometres. Crops grown include cotton, millet, sorghum, sweet potatoes, simsim. The Total population in Amuru district is 186,696, total households are 36,650 [43].

### Data collection and selection of the informants

A cross-sectional study was conducted between September 2020 and April 2021. Three districts were purposively selected based on rich biodiversity of nutraceutical plant species and presence of TMPs.

Study population were all people from Amuru, Gulu and Pader districts who have used nutraceutical plants for 1 year or more to acquire adequate knowledge on herbal medicine [12].

### Sampling strategies

In this research, four hundred TMPs were purposively selected and interviewed from Gulu, Amuru and Pader districts, Northern Uganda. The sample size was determined using the Yamane formula [47] (1) with 95% confidence level presented as follows:

1 $$n = \frac{N}{{1 + N\left( e \right)^{2} }}$$

where *n* is the sample size required = 400. *N* is the total number of people in the study area (739,700) [43]. *e* is the maximum variability or margin of error 5% (0.05). 1 is the probability of the event occurring.

Sample size included one hundred and thirty three (133) people from Amuru district, 133 from Gulu district and 134 from Pader district. With Prior Informed Consent (PIC), Purposive sampling [48–50] was used to select 400 authentic and well-known traditional medicine practitioners (TMPs) who included key informants, such as professional herbalists, herbal farmers and herbal sellers. Professional herbalists owned herbal ‘clinics’, where they diagnosed and treated people for money, herbal farmers owned gardens, where they grew nutraceutical plants, while herbal sellers bought nutraceuticals in bulk and sold retail in the market. Subsequent respondents were identified using snowball sampling method [48, 51]. Respondents were identified with the help of local government officers, particularly local councillors LC I, LC II, and LC III heading village, parish and sub-county levels, respectively.

Data were collected using semi-structured interviews, focus group discussions and direct observation. The prior-informed consent (PIC) form was translated into the local Acholi language. The scope, possible benefits and risks of the study were explained to willing TMP interviewees and Focus Group participants who signed consent forms after agreement. Identification of interviewees and Focus Group participants were protected via anonymisation.

The TMPs were asked whether they were familiar with signs and symptoms of HIV/AIDS and associated opportunistic infections. TMPs were asked how they assumed a person probably has HIV and if they relied on biomedical lab diagnoses for confirming the patient’s HIV sero status or depended on confession by the patient. The TMPs were asked if they treated patients already receiving ARVs. Informants were not required to reveal HIV/AIDS status to researcher.

During the conversation, data on respondent characteristics and information related to Nutraceutical uses of plants for the management of HIV/AIDS-related diseases were captured. Interviews were conducted at the herbalist ‘clinic’, at market place, where some nutritious plants sold, at the home of respondents and also during field excursions to point out the plants. This information included a complete list of local names of plants, plant parts used to treat HIV/AIDS Opportunistic infections, methods of preparation, route of administration and dosages were recorded. According to US Centres for disease control [52], the major opportunistic infections associated with HIV/AIDS are candidiasis, pneumonia and tuberculosis, which were considered in this study. Other symptomatic but undefined conditions considered included diarrhoea, cough, stomach ache, skin fungal infection and venereal diseases [53]. Nutrition-related conditions were considered, such as anaemia, low appetite for food and low immunity. Focus Group Discussions (FGD) used the Participatory Action Research (PAR) Model [54, 55]. TMPs such as professional herbalists, herbal farmers and herbal sellers participated in the FGD. Approximately eight to ten (8–10) participants in each FGD were involved [54, 55]. FGD is where the researcher assembled a group of individuals to discuss a specific topic, aiming to draw from complex personal experiences, beliefs, perceptions and attitudes of participants through a moderated interaction [56]. Four FGD per district were conducted. FGD were conducted at a central convenient meeting point, such as under a tree or a shelter.

FGD answered clear and simple questions set to elicit information on Quality Assurance and Quality Control of nutraceutical plant preparation. The questions intended mainly to understand the Acholi cultural context of Quality control methods employed in the management of Opportunistic infections using Nutraceutical plants. Information on harvesting equipment, utensils used, source of water and fuel or location of plants was got by direct observation.

Fresh plant materials were collected from forests, bushes or people’s herbal medicine gardens by this researcher (trained botanist/taxonomist) and a research assistant using standard procedures [57, 58] and the practitioners were used as guides in field excursions. Plant specimens were collected based on ethnomedical information provided by TMPs and were identified in the field basing on the African Plant Database and Flora of Tropical East Africa catalogue [59]. Plant names were checked and updated with online website (www.theplantlist.org). The voucher plant specimens were pressed, dried, mounted, coded [57, 58] and deposited at the Herbarium in the Department of Plant science, microbiology and Biotechnology, Makerere University Kampala, Uganda. Further confirmation and identification of botanical specimens was done via comparison with herbarium material stored in the Makerere University Herbarium and consultation with curators [57, 58]. Accession numbers of plant species were recorded.

### Data analysis

Collected Ethnobotanical data were entered into Microsoft Excel spreadsheet 2013. Descriptive statistics (graphs and tables) was used to summarize ethnobotanical and socio-demographic data. All recorded plant species are presented in tabular format, alongside corresponding ethnomedicinal usage information. The number of nutraceutical plants cited per TMP was used as a proxy for the extent of their traditional knowledge.

Differences in traditional nutraceutical knowledge due to gender, age, level of education, religion and location were analysed using analysis of variance (one-way ANOVA) test and independent *T* test. Number of nutraceutical plants reported set as the dependent variable, while gender, age, level of education, religion, location set as the independent variable. A Linear Regression model was used to understand whether age and time in schooling system predict nutraceutical plant knowledge. Qualitative data analysis used Thematic Content Analysis technique [37, 60–62] and narrative analysis [63, 64] to understand traditional methods used during collection, processing and storage of nutraceuticals as an indicator of quality control.

Quantitative data were analysed using the familiarity index, Fidelity level and informant consensus factor [65].

The familiarity index (FI), a relative indicator of the familiarity of a plant species is defined as the frequency a given plant is mentioned as an ethnomedicine divided by the total number of knowledge holders or TMPs interviewed in the study [65].

### Fidelity level

Fidelity level (FL) values indicate most preferred plant species for particular ailments [65].

F_L_ = I_P_/N × 100, where I_P_ is the number of informants who indicated use of a species for the same major ailment, N is the total number of informants who mentioned the plant for any other use. Increasing values of FL for a species indicate its uniqueness to treat a particular illness.

### Informant consensus factor

The Informant consensus factor (ICF) is the number of citations in each ailment category minus the number of species used, divided by the number of use citations in each category minus one. The ICF value indicates the Level of Agreement among Herbalists in the use of plants for various ailments [65]. F_ic_ = (N_ur_ − N_t_)/(N_ur_ − 1), where N_ur_ is the number of use reports of informants for particular ailment/disease category, N_t_ is the number of plant species used for a particular ailment category by all informants.

Informant consensus factor is performed to test the consistency of the informants’ knowledge in treating a particular opportunistic infection caused by bacteria and fungi. ICF values range between 0 and 1, where 1 indicates the highest level of informant agreement. Ethnomedicinal use were classified by body systems according to disease categories proposed by the International Classification of Diseases [53].

## Results

### Profile of traditional medicine practitioners

**Fig. 1:**
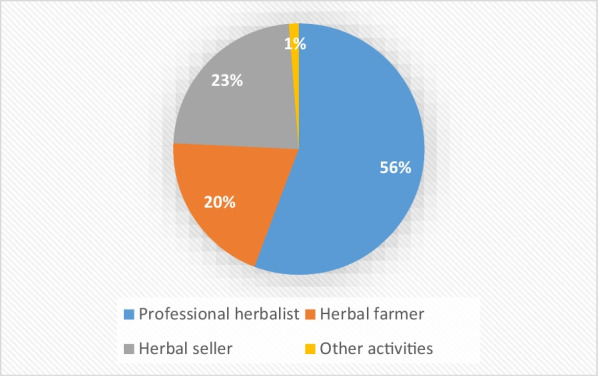
Profession of informants

Results indicate that Traditional healing is widely acknowledged as an occupation in Northern Uganda. Of the four hundred (400) respondents, 55.75% were Professional Herbalists, 20% regarded themselves as herbal farmers and 23% were herbal sellers (Fig. 1). Some informants were involved in other activities, such as hairdressing, teaching, and tailoring.

The profile of informants regarding age, gender, education, religion, location is shown in Table 1. Nutraceutical plant knowledge was significantly influenced by gender, age, and location (*p* ≤ 0.05) (Table 1). Both men and women used nutraceutical plants to manage opportunistic infections associated with HIV/AIDS (Table 1). Ninety-four point five percent (94.5%) of the TMPs were women and 5.5% men (Table 1). The mean number of Nutraceutical plants used to manage opportunistic infections associated with HIV/AIDS, cited by women (3.608 plants) showed great extent of their traditional knowledge than the men (2.454 plants) (*p* ≤ 0.05) (Table 1).

**Table 1:** Nutraceutical plant knowledge among different social groups of the study area

Parameter	Informant group	*N* (sample size)	Mean no. of plants cited	T statisics	*p* value	Sum of squares SS (total)	Degree of freedom df (total)	*F* value	*p* value	*F* critical
Gender	Male	22	2.454	2.748	0.011	1955.19	399	5.7160	0.017275	3.8649
	Female	378	3.608							
Age (years)	Below 21	23		2.352	0.011	818.1	170	4.848	0.001008	2.4260
	21–35	170								
	36–50	103	3.365 (adult)							
	51–65	67	4.058 (elder)							
	above 65	37								
Religion	Catholic	344				542.57	83	2.1018	0.1065	2.7188
	Muslim	1								
	Protestant	27								
	Born-again	28								
Education level	no formal education (illiterate)	58	3.948 (iliterate	1.463	0.147	69.69	12	0.4311	0.7829	3.8379
	Primary school	324	3.476 (literate)							
	Completed O’Level	14								
	Completed A’Level	3								
	Diploma	1								
	Degree	0								
Location	Gulu	133				1955.19	399	6.0810	0.002504	3.0185
	Amuru	133								
	Pader	134								

Evaluation of the influence of age indicated that few TMPs were in the age group of the youth, where 5.75% were below age 21 years (Table 1). Forty-two point five percent (42.5%) were between ages 21–35% and 25.75% between ages 36–50 years (Table 1). Most of the people who had substantive knowledge on Nutraceutical plants (4.058 plants) were mostly older than 50 years of age (Table 1). The mean number of Nutraceutical plants used to manage opportunistic infections associated with HIV/AIDS, cited by Elders (above 50 years) (4.058 plants) was higher than that cited by adults (3.365 plants) and this showed the great extent of elders’ traditional knowledge. TMPs’ knowledge on nutraceutical plants grows during their lifetime as seen from the Linear Regression Analysis which showed that Nutraceutical plant knowledge and age had a Positive significant Correlation (*R*^2^ = 0.0524, *p* ≤ 0.01) (Table 2). Linear regression was used for time series forecasting. Age and level of education change with time unlike gender and religion which do not change with time.

**Table 2:** Age and level of education predicting knowledge of nutraceutical plants used to manage opportunistic infections associated with HIV/AIDS

Parameter	*R*-square value	*F* value	*p* value
Age	0.0524	22.028	0.0000037
Level of education	0.0038	1.527	0.217200

As regards the Education level of the TMPs, fourteen point five percent (14.5%) were illiterate and most of the TMPs had incomplete fundamental education, where 81% reported attending Primary school (Table 1). Although the illiterate TMPs had higher knowledge on nutraceuticals than TMPs who attended primary school, the difference in plant knowledge was not significant. (*p* > 0.05) (Table 1). Results from Linear Regression analysis indicate that the level of education attained had a significant negative correlation with Nutraceutical plant knowledge (*R*^2^ = 0.0038, *p* ≤ 0.01) (Table 2). Nutraceutical plant knowledge was not significantly influenced by education as most respondents had incomplete fundamental education (*p* ≥ 0.05) (Table 1). Respondents belonged to different religions (Table 1).

### Diversity of neutraceutical plants, their use, and growth forms

This ethnobotanical study recorded a total of eighty four (84) nutraceutical plant species distributed in 38 plant families and 76 genera, used to manage opportunistic infections associated with HIV/AIDS (30 of the 84 plants are shown in Tables 3, 4, and 5) (See “Appendix” for the 54 plants). Most abundant families were Leguminoceae (13 species), Asteraceae (6 species), Solanaceae (4 species), Verbenaceae (4 species) and Moraceae (4 species).

**Table 3:** Nutraceutical plants used to manage bacterial opportunistic infections associated with HIV/AIDS and mode of preparation for quality control

Scientific name, family, Local name in native “Acholi dialect”	Accession number	Habit	Habitat	Conservation status	Bacterial opportunistic infection treated (uses)	Part used	Mode of preparation and administratn	Familiarity index	Fidelity level
*Acacia hockii* De Wild *(*Leguminoceae) Okuto-oriang	MHU 32118	Shrub	Bu	W/A	Cough. Diarrhea, stomachache	FL	Mix leaves in hot water, drink infusion	0.058	0.115
*Aloe vera* L*.* Aloaceae Ataka-rach	MHU 50969	Herb	Ho	Cu/A	Cough. Stomachache, diarrhea	S	Mix sap from leaves in cold water, drink	0.102	0.205
*Bidens pilosa* L Asteraceae Labika	MHU 51149	Herb	Rs	W/A	Cough, wounds, eye infection, ear infection	FL	Mix leaves in hot water, drink infusion	0.075	0.006
*Bridelia scleroneura* Mull.-Arg Phyllanthaceae Larwece	MHU 35756	Shrub	Bu	W/A	Diarrhea, cough.eye infection	FSB, FR	Boil bark or roots, drink decoction	0.065	0.26
*Cajanus cajan* L.Millsp*.* Leguminoceae Lapena	MHU 51151	Shrub	Ga	Cu/A	Cough. Diarrhea	FL	Chew Leaves	0.20	0.80
*Cassia nigricans* Vahl Leguminoseae Abanceng	MHU 32957	Shrub	Bu	W/A	Abdominal pain, cough, sore throat	FL	Mix leaves with hot water, drink infusion	0.045	0.09
*Combretum collinum* Fresen Combretaceae Oduku	MHU 34092	Tree	Fo	W/A	Diarrhea, stomachache, wounds	FR, FL	Boil roots or leaves, drink decoction	0.073	0.097
*Eucalyptus globulus* Labill Myrtaceae Kalatuc	MHU 51152	Tree	P	Cu/A	Cough	FL	Mix Leaves in hot water, drink infusion	0.203	0.405
*Euphorbia tirucalli* L Euphorbiaceae Kilajok	MHU 50984	Shrub	Bu	W/A	Eye infection, cough, eye infection, diarrhoea	S	Squeeze sap into eye	0.063	0.250
*Ficus sycomorus* L Moraceae Olam	MHU 37779	Tree	Bu	W/A	Syphilis, diarrhea, stomachache	FL,FSB	Boil leaves or bark in water, drink decoction	0.048	0.011
*Mangifera indica* L Anacardiaceae Muyeeme	MHU 41712	Tree	Ho	Cu/A	Cough, diarrhea, stomachache	FSB	Boil bark, drink decoction	0.188	0.750
*Momordica foetida* Schumach Cucurbitaceae Bomo	MHU 51150	Climber	Bu	W/A	Syphilis, diarrhea, stomachache	FR	Boil roots, drink decoction	0.075	0.008
*Piliostigma thonningii* (Schumach.) Milne.Redh Leguminaceae Ogali	MHU 35791	Tree	Bu	W/A	Cough, wounds, bloody diarrhea, stomachache	FL	Mix leaves in hot water, drink infusion	0.093	0.09
*Solanum incanum* L Solanaceae Ocok	MHU 32003	Shrub	Rs	W/A	Stomachache, ear infection, sore throat	FR,FL	Boil roots or leaves, drink decoction	0.078	0.310
*Vernonia lasiopus* O.Hoffm Asteraceace Labuka	MHU 35754	Shrub	Bu	W/R	Eye and ear infection, stomachache	S	Squeeze sap into ear, eye	0.048	0.095

**Parts used**: **FL**: fresh leaf, **FR**: fresh root, **FSB**: fresh stem bark **FSe:** fresh seed, **FFr**: fresh fruit, **Bu**: bulb, **S**: sap

**Habitat: Bu**—bushland, **Gr**—grassland, **Ho**—homestead, **Rs**—roadside, **P**—plantation **Sw**—swamp, **Ga**—garden, **Fo**—forest, **Wo**—woodland

**Conservation status**: **W/A**: wild and abundant, **W/R**: wild and rare, **Cu/A**: cultivated and abundant, **Cu/R**: cultivated and rare

**Decoction**: water extraction by boiling of dissolved plant material and drank

**Infusion**: plant material is added to hot water and left to stand for few minutes and drank

**Table 4:** Nutraceutical plants used to manage fungal opportunistic infections and mode of preparation for quality control

Scientific name, Family, Local name in native “Acholi dialect”	Accession number	Habit	Habitat	Conservation status	Fungal opportunistic infection (uses)	Part used	Mode of preparation	Familiarity index	Fidelity level
*Afromomum angustifolium* (Sonn.) K. Schum Zingiberaceae Oceyu	MHU 32633	H	Bu	W/R	Oral candidiasis	FL	Boil leaves, drink decoction	0.020	0.026
*Erigeron floribundus* (Kunth) Sch.Bip Asteraceae Dingtong	MHU 51148	H	Rs	W/A	Skin fungal infection	FL	Rub leaves on skin with shea butter	0.075	0.021
*Ficus sycomorus* L Moraceae Olam	MHU 37779	T	Bu	W/A	Skin fungal infection	S	Rup sap on skin	0.045	0.011
*Gynandropsis gynandria* (L.)Briq Cleomeaceae Akeyo	MHU 35837	H	Ga	Ga	Oral candidiasis, skin fungal infection	FL	Boil leaves, drink decoction	0.025	0.006
*Khaya senegalensis* (Desr.). A. Juss Meliaceae Tido	MHU 42488	T	Bu	W/A	Oral candidiasis, candida	FL	Boil bark, drink decoction	0.053	0.019
*Momordica foetida* Schumach..Cucurbitaceae Bomo	MHU 51150	C	Bu	W/A	Oral candidiasis, candida	FR	Boil roots drink, decoction	0.090	0.017
*Occimum gratissimum* Linn.Lamiaceae Mida	MHU 35677	H	Bu	W/A	Skin fungal infection	FL	Rub leaves on skin	0.050	0.040
*Pseudocedrela kotschyi* (Schweinf.) Harms Meliaceae Ofuti	MHU 50947	T	Bu	W/R	Oral candidiasis	FR	Boil roots, drink decoction	0.028	0.037
*Sanseviera neutoniana* T.G Forrest Asparagaceae Tworo-gwok	MHU 42552	H	Bu	W/R	Oral candidiasis	FL	Boil leaves, drink decoction	0.025	0.025
*Sarcocephalus latifolius (Smith)*Rubiaceae munyu)	MHU 50529	S	Bu	W/A	Oral candidiasis	FL	Boil roots, drink decoction	0.020	0.027

**Parts used**: FL: fresh leaf, FR: fresh root, FSB: fresh stem bark, FSe: fresh seed, FFr: fresh fruit, Bu: bulb, S: sap

**Habitat:** Bu—bushland, Gr—Grassland, Ho—homestead, Rs—roadside, P—plantation, Sw—swamp, Ga—garden, Fo—forest, Wo—woodland

**Habit**: S—shrub, T—tree, H—herb, C—climber, G—grass

**Conservation status**: W/A: wild and abundant, W/R: wild and rare, Cu/A: cultivated and abundant, Cu/R: cultivated and rare

**Decoction**: water extraction by boiling of dissolved plant material and drank

**Infusion**: plant material is added to hot water and left to stand for few minutes and drank

**Table 5:** Nutraceutical plants used to boost immunity against opportunistic infections and mode of preparation for quality control

Scientific name Family, Local name (Ethnic language) in native “Acholi dialect”	Accession number	Habit	Habitat	Conservation status	Use	Part used	Mode of preparation	Familiarity index
*Amaranthus dubius* Mart.ex Thell Amaranthaceae Obuga	MHU 42023	Herb	Ga	Cu/a	Immune booster	FL	Decoction leaves	0.005
*Bidens pilosa*. L Asteraceae Labika	MHU 51149	Herb	Rs	w/a	Increases CD4/wound healing properties/blood clotting	FL	Decoction leaves	0.005
*Carica papaya* Linn Caricaceae Papai	MHU 42266	Shrub	Ho	Cu/a	Immune booster	FL/FFr	Decoction leaves	0.003
*Cucurbita pepo* L Cucurbitaceae Okono	MHU 42492	Climber	Ga	Cu/a	Increases appetite immune booster	FL/FFr	Decoction leaves	0.013
*Vigna unguiculata* L. *(Walp)* Leguminoseae Boo	MHU 35800	Herb	Ga	Cu/a	Immune booster	FL	Decoction leaves	0.005

**Parts used**: FL: fresh leaf, FFr: fresh fruit

**Habitat:** Bu—bushland, Gr—grassland, Ho—homestead, Rs—roadside, P—plantation, Sw—swamp, Ga—garden, Fo—forest, Wo—woodland

**Habit**: S—shrub, T—tree, H—herb, C—climber, G—grass

**Conservation status**: W/A: wild and abundant, W/R: wild and rare, Cu/A: cultivated and abundant, Cu/R: cultivated and rare

**Decoction**: water extraction by boiling of dissolved plant material and drank

**Infusion**: plant material is added to hot water and left to stand for few minutes and drank

**Fig. 2:**
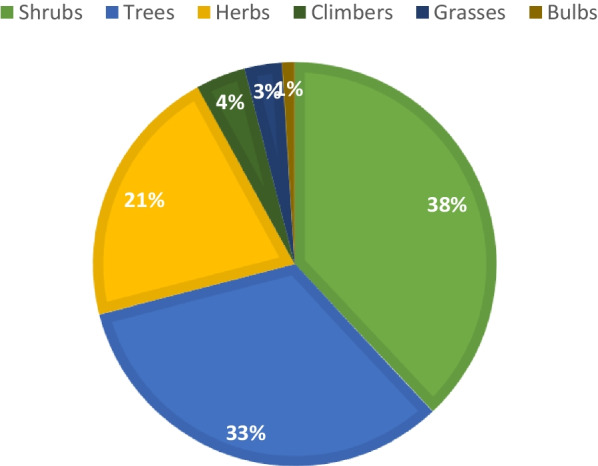
Growth form of nutraceutical plants

High Familiarity Index values (FI) and high-fidelity level (FL) values show outstanding preference for treating opportunistic infections (Tables 3, 4 and 5). Six nutraceutical plants that had higher fidelity level and familiarity index values included *Momordica foetida* Schumach, *Erigeron floribundus* (Kunth) Sch. Bip, *Mangifera indica* L, *Cajanus cajan* L. Millsp, *Eucalyptus globulus* Labill and *Cucurbita pepo* L. Plant growth forms (habits) analysis indicated that 38% were shrubs and 33% were trees. Herbs comprised 21% of total number (Fig. 2).

**Fig. 3:**
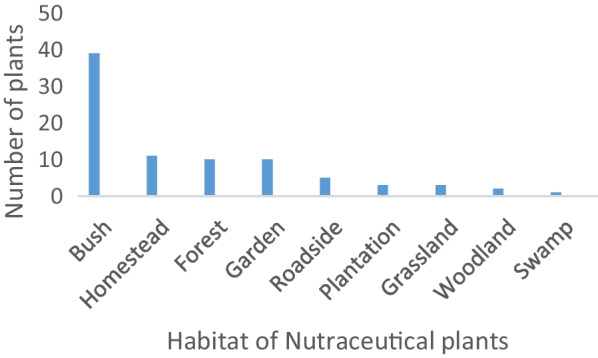
Source of nutraceutical plants

Majority of plants were collected from the bushes (47%) and 13.8% cultivated in gardens, and 13% were got from around homestead and forests (9.8%). Others were obtained from roadsides (7.3%), plantations (3.3%) or woodland (2.4%) (Fig. 3).

### Methods of preparation, administration and ailments treated

Decoctions/boiling in water (70%) were commonly used as mode of preparation followed by infusion/mixing with hot water (25%), maceration/soaking in cold water (3%), and others (2%) (Tables 3, 4, 5). Routes of administration included oral (76%), dermal (17%), auricular (3%), ocular (2%) and others (2%) (Tables 3, 4, 5).

The most preferred plant species for bacterial opportunistic infections include *Cajanus cajan* leaves (for cough, tuberculosis, chest infections) (FI = 0.20, FLI = 0.80), *Mangifera indica* Bark (for diarrhoea and stomach-ache) (FI = 0.19, FLI = 0.75) and *Eucalyptus globulus* leaves (FI = 0.20, FLI = O.41) (for cough, tuberculosis, chest infections) (Table 3).

The most preferred plant species for Treating Fungal opportunistic infections include *Momordica foetida* roots (For oral candidiasis) (FI = 0.090, FLI = 0.017) and *Erigeron floribundus* leaves (for skin fungal infection) (FI = 0.075, FLI = 0.021) (Table 4).

Most preferred Nutraceutical plants that are used to boost immunity were also noted (Table 5). The percentage of total number of plants that provided nutritional support in boosting immunity was 12% (Table 5).

The Informant Consensus Factor (ICF) indices indicated that the highest level of herbalists’ agreement on the nutraceutical plants used to manage bacterial and fungal infections was for Digestive system, Respiratory system and Dermatological systems with ICF Values of 0.88, 0.87 and 0.85, respectively, as shown in Table 6.

**Table 6:** Informant consensus factor for six categories of ailments

Body system according to disease category	Number of use reports (N_ur_)	Number of plant species used (N_t_)	Informant consensus factor F_ic_ = (N_ur_ − N_t_)/(N_ur_ − 1)
Digestive system (mouth, oesophagus, stomach, small and large intestine, rectum, anus)	610	74	0.88
Respiratory system (nose, trachea, bronchi, lungs, diaphragm)	326	38	0.87
Dermatological system (skin)	106	17	0.85
Nervous system (brain, eye, ear. Spinal cord)	103	25	0.76
Reproductive system	11	9	0.20

Thematic content analysis and narrative analysis of responses by TMPs about the prioritized problems encountered during harvest, processing, production and administration of the documented nutraceutical plants, the local solutions to prioritized problems and recommendations to the government on opportunities to help with quality control measures are shown in Table 7.

**Table 7:** Challenges encountered during harvesting, processing and administration of nutraceuticals and recommendations to authorities

Theme	Sample narrative	Recommendation
Problems encountered during collection of nutraceutical plants	**Distance to source of nutraceuticals** *‘Long distance walking to forest, bush to find nutraceuticals’* **Safety of TMPs during collection** *‘We meet wild animals, get pricked by thorns, sharp objects in bush ‘* **Availability of protective gear** *‘we lack gumboots’*	‘We ask Government of Uganda to facilitate with gumboots and protective gear’
Problems encountered during processing of nutraceutical plants	**Availability of equipment** *‘we lack processing equipment such as grinding machines’* *‘Crushing or grinding is done using two rough stones or mortar and pestle. Utensils used are clay pots or Aluminium/steel saucepans’* *It was observed that during processing, nutraceutical plant materials had visible signs of contamination, while some equipment used were not clean. TMPs engaged in herbal processing did not maintain hygiene or wear protective clothing*	‘We ask Government of Uganda to facilitate with crushing equipment’
Problems encountered during preservation of nutraceuticals	**Methods of preservation** *‘we don’t preserve nutraceuticals, plants are abundant’* *‘we have limited knowledge on preservation of herbal formulations’*	‘We need training’
Problems encountered during packaging nutraceuticals	**Packaging of nutraceuticals** *‘we lack packaging materials ‘*	
Problems encountered during treatment of clients	**Methods of treatment** *‘we estimate doses for nutraceuticals* *‘We treat opportunistic infections till no symptoms seen’* **Cooperation with medical doctors** *‘there is Lack of cooperation with medical doctors’*	‘we ask for collaboration and communication between biomedical clinicians and Traditional medicine practitioners should be encouraged
Problems encountered during payment for nutraceuticals	**Payment for herbal medicine** *‘payment is low or is made inkind in exchange for chicken or harvested crops’*	Ministry of Education, Uganda is called upon to convene and train TMPs about basic knowledge on diagnosis of disease, record maintenance, Legal systems, business management, processing and packaging nutraceutical plants for commercial use, how to maximize profits and protect their knowledge
Problems encountered in keeping information	**Methods of storing information** *‘Most TMPs cannot read or write especially the elderly, therefore, unable to record information’* *‘Information passed orally from generation to generation’*	

Most TMPs reported that they had limited knowledge on preservation of herbal formulations and most times used freshly collected nutraceutical plant parts, because most plants are still abundant (Table 7).

When consulted by a patient, the TMPs interview patient orally about their problem and history. Some patients bring along their medical diagnosis from hospitals. Symptoms of various HIV/AIDS opportunistic infections are described to the healers so as to enable them give the appropriate plant species they usually use to manage infections. Doses were measured using 0.5 L plastic cups or stainless-steel tablespoons but there was ambiguity on how this was applied (Tables 3, 4, 5, 6, 7). Duration of treatment depended on time of disappearance of symptoms and was not specific but varied from herbalists to herbalist. Time of treatment depends on when one recovers. Estimates of doses is not uniform and depend on age, and type of infection. Most of the TMPs did not keep record on infections treated or nutraceutical plants used (Table 7). All TMPs had insufficient knowledge about packaging and presentation techniques. Transmission of knowledge to trainers of nutraceutical plant use was mainly informal (Table 7).

## Discussion

### Profile of traditional medicine practitioners

Although both men and women use nutraceutical plants to manage opportunistic infections associated with HIV/AIDS, women showed great extent of their traditional knowledge than men (Table 1). These results are in agreement with several other reports which found the same tendency in their studies about nutraceutical plant knowledge [66–68], in that women knew more about nutraceutical plants than men. The predominance of women, could be because women had double role as the nutritional food providers and family primary health caregivers during an illness, this may explain their expertise in nutraceutical plant use. Cheikhyoussef et al. [69], observed that traditional healing is a gender-based practice. Results in this study are similar to reports from Jeddah, Saudi Arabia, where preference for medicinal plant use is dependent on gendered socio roles and experience [70]. Gender role analysis by Singhal [71] in relation to medicinal plants revealed that collection, processing, storage, utilisation of medicinal plants is mainly assigned to women.

Although both adults and elders (above 50 years) use nutraceutical plants to manage opportunistic infections associated with HIV/AIDS, elders (above 50 years) showed great extent of their traditional knowledge than adults (Table 1). These results are consistent with those obtained by [72–74], where diversity on nutraceutical plant species and uses cited among the young people were on average lower than number cited by the elderly. Ouhaddou et al. [75] also reported that elderly herbalists, above 50 years of age, have more knowledge on medicinal plants with regard to other age groups. Reasons for greater knowledge could be that with progressive age, people have more time to accumulate knowledge and, therefore, show greater nutraceutical plant knowledge and the lesser knowledge in the younger population is because of the ongoing socio-economic and cultural changes [72].

Although there was no significant difference in nutraceutical plant knowledge between the illiterate and literate respondents (p>0.05), the mean number of plants cited by illiterate TMPs was higher than those of their literate counterparts (Table 1). These results are consistent with those obtained by [66, 73, 76], who observed that nutraceutical plant species and uses cited among the educated are on average lower than the number cited by the illiterate. Weckmuller et al., [66] observed that among Waorani society, Equador, formal education programs marginalise indigenous knowledge on nutraceutical plants by encouraging an urban lifestyle that leads to lack of interest in nutraceutical plants. Bruyere et al., [76] proposes that communities undergoing change should develop options to transmit traditional knowledge on nutraceutical plants to its younger population who are undertaking formal education and lack interest in nutraceutical plant knowledge. Respondents belonged to different religions (Table 1). Religion teaches people moral behaviour, provides support during depression, anxiety and offers guidance with coping skills during an illness [77].

### Diversity of nutraceutical plants, their use, and growth forms

This Ethnobotanical study recorded eighty four (84) nutraceutical plant species distributed in 38 plant families and 76 genera, used to manage opportunistic infections associated with HIV/AIDS in Gulu, Amuru and Pader districts. Most abundant families were Leguminoceae, Asteraceae, Solanaceae. Family Leguminosae contain alkaloids, amino acids, cynogenetic glucosides, anthocyanins, tannins, flavonoids [79]. Asteraceae contain terpenes/terpenoids, carboxylic and fatty acids [80], while Solanaceae contain solanine, tomatidine, capsaicin [81]. Shrubs were mostly used, because they are easily found and can withstand semi-arid climate while taking shorter time to mature than trees [78]. Most of the nutraceutical plants were found in bushes, where they were wild and abundant [45]. Al-Obaidi et al. [78] agrees that medicinal shrubs are easily available than herbs, because they can grow in arid and semi-arid areas. The bioactivity of nutraceutical plant shrubs could be attributed to the presence of secondary metabolites [79–81].

### Methods of preparation, administration and preservation

Leaves were most frequently used for preparation of medicine, followed by bark and roots (Tables 3, 4, 5). The bioactivity of these leaves, bark and roots of nutraceutical plant could be attributed to the presence of secondary metabolites. The frequent use of leaves, bark and roots was also reported by [45], who observed unsustainable harvesting techniques of nutraceutical plants in Northern Uganda.

Decoctions/boiling in water (70%) were commonly used as mode of preparation and oral route (76%) was most used route of adminstration. Majority of preparation was made using water as a medium, as also reported in studies by [87, 88]. According to Tugume et al. [67] boiling in water is effective in extracting plant materials and preserving remedies for a longer period compared to maceration in water. Saikia et al. and Mesfin et al. [87, 88] also reported that most common route of administration of herbal preparation was oral route. According to Kim and De Jesus [88] the oral route of administration is a convenient, cost-effective and most commonly used drug administration route. The primary site of drug absorption is usually the small intestine and the bioavailability of the drug is influenced by the amount of drug absorbed across the intestinal epithelium [88].

The number of nutraceutical plants recorded in the study area is lower than medicinal plants recorded by Anywar et al. [13] (*n* = 236), but comparable to the the number of medicinal plants recorded by Mugisha et al. [82], (*n* = 81), Tahir et al. [31] (*n* = 103) Lamorde et al. [39] (*n* = 103) and Shehu et al. [32] (*n* = 52). These ethnobotanical studies, indicate that the majority of nutraceutical and aromatic plants come from wild sources and is the source of livelihood for millions of people. Ethnobotanical studies facilitate participation of an ethnic people in the collection and assessment of botanical knowledge through field studies, which give valid information about the utility of plant species [83–85].

### Ailments treated

#### The antibacterial properties of *Eucalytptus globulus *Labill, *Cajanus cajan* L. Millsp and Mangifera indica L.

The leaves of *Eucalytptus globulus* Labill (known as *Kalatuc in Acholi*) are used for treating cough, tuberculosis, and chest infections. Bachir and Benali [89] reported that essential oil in leaves of *Eucalyptus globulus* has antimicrobial activity against bacteria. Alvarenga et al. [90], also reported thirty two (32) airbone anti-Tuberculosis components were identified in *Eucalyptus citriodora*. Phytochemical analysis of leaf extract of *Eucalyptus globulus* proved the presence of tannins, saponins, terpenoids, glycosides, alkaloids, phenolic compounds, cardiac glycosides, terpenes, reducing sugars, acrbohydrates, flavonoids [91].

The leaves of *Cajanus cajan* L. Millsp (also known as pigeon pea—English, Lapena—Acholi), are used to treat cough, tuberculosis, chest infections. *Cajanus cajan* is an important grain–legume food crop with high levels of proteins [92]. Saxena et al. [93] reported that Pigeon pea *Cajanus cajan* is capable to prevent and cure bronchitis, cough, pneumonia and respiratory infection. Oke [94] reported *Cajanus cajan* leaves to contain alkaloids, flavonoids, tannins, saponins, terpenes, phlobatannins, anthraquinones and sterols.

The tree bark of *Mangifera indica* L. (known as Mango—English, Muyeeme—Acholi) are used for treating Diarrhoea, dysentry and stomach-ache. Study by Osei-Djarberg et al. [95] showed that bark and leaf extracts of *Mangifera indica* has antimicrobial activity. Sanusi et al. [96] reported that the antimicrobial activity obtained in their study support the claim by the local communities for the use of *Mangifera indica* stem bark decoction for treatment of infections, such as diarrhoea. Phytochemical screening of crude stem bark extracts of *Mangifera indica* revealed the presence of tannins, saponins, alkaloids, flavonoids, cardiacglycosides and phytosterols [96].

#### The antifungal properties of *Momordica foetida *Schumach and *Erigeron floribundus* (Kunth) Sch. Bip

The roots of *Momordica foetida* Schumach (known as Bitter cucumber—English and Bomo—Acholi) are used to treat genital and oral candidiasis. Our results are supported by findings of [44] who reported that commonest plant used to manage candidiasis in Northern Uganda was *Momordica foetida*. Muronga et al. [97] found *Momordica foetida* to contain proteins, fibres, terpenoids, glycocides, alkaloids, flavonoids, tannins, phenolic compounds, gallic acid, phenolic glycosides, steroids, cardiac glycosides, phenolics, saponin, carotene.

*Erigeron floribundus* (Kunth) Sch. Bip (Dingtong—Acholi), leaves used for treating skin fungal infection. Berto et al., [98] reported that *Erigeon floribundus* is used for treatment of fungal skin infections and Candida. Bi et al. [99], observed antifungal activity of *Erigeron floribundus* against a wide range of dermatophytes. Moungang et al. and Dall’Acqua et al. [100, 101] found *Erigeron floribundus* to contain alkaloids, saponins, polyphenols, tannins, cardiac glycosides, flavonoids, quinones.

### The immune boosting properties of *Cucurbita pepo* L.

*Cucurbita pepo* L. (Pumpkin—English, okono—Acholi) leaves was widely used for immune boosting. These results are consistent with a study by Almohaimeed et al. [102], were *Cucurbita pepo* L. enhanced wound healing process in rats through the antioxidant, anti-inflammatory and anti depressant activities.

Other nutraceutical plants recorded in this study were reported elsewhere. *Albizia coriaria* Oliv. is widely used in traditional medicine for treating opportunistic infections, such as skin infections, cough, syphilis, and sore throat among PLHIV in Uganda [12, 40]. *Erythrina abyssinica* Lam. has been reported to be used to treat tuberculosis in Uganda [103]. The root of *Serudaca pedunculata* Fresen. is commonly used in Uganda for treating STDs, T.B, and Coughs [12]. Maud et al. [104] noted that *Hibiscus sabdariffa L*, *Plumeria obtuse L* and *Abutilon guineense* were most frequently used by immune-compromised people with HIV/AIDS in Western Uganda. *Acacia hockii* used to manage opportunistic infections as reported by [36, 105]. A study conducted in Mieso district, eastern Ethiopia by [106] reported that of the 41 wild edible plant species, 13 species had medicinal value.

Analysis of informant consensus factor (ICF) indicated that the highest level of herbalists’ agreement on the nutraceutical plants used to manage bacterial and fungal infections was for Digestive system, Respiratory system and Dermatological systems with ICF Values of 0.88, 0.87 and 0.85, respectively, as shown in Table 6. These plants become target in efficacy tests [107, 108]. This determination of Informant consensus factor among local communities helps to evaluate drugs of herbal origin [109].

### Conservation status of nutraceutical plants

Plant parts such as roots, bark, leaves, fruits or sap were harvested from bush, forest or garden (Tables 3, 4, 5). Most of the nutraceutical plants in study area are wild and abundant (Tables 3, 4, 5). These results are consistent with Uganda National Environment Management Authority report [42], where approximately 5000 species of higher plants in Uganda were recorded, of which 70 are endemic [42]. However, high percentage use of plant roots and bark threatens the sustainable use from the wild. The TMPs confirmed decrease in the richness and abundance of some plant species. The high nutraceutical plant species richness and species diversity in Northern Uganda needs to be protected [45]. Akwongo et al. [44] observed that unsustainable harvesting techniques in Northern Uganda, like uprooting plants may lead to loss of plant species diversity and indegenous knowledge. Oryema et al. [45], noted that plants used to manage tuberculosis in Northern Uganda faced conservation threats due to exploitation of roots.

### Problems faced during quality control of nutraceuticals

Methods observed for quality control may not guarantee safety of use of these nutraceuticals (Tables 7). According to WHO [19] environmental pollution, misidentification and contamination all affect safety and quality of nutraceutical plants products. During collection, nutraceutical plant materials should be free from visible signs of contamination by insects, moulds, animal excreta. Any soil, sand, stones and other foreign inorganic matter should be removed before nutraceutical plants are cut or ground for testing [19]. No poisonous or dangerous foreign matter or residue allowed and no abnormal odour or discolouration allowed [19]. All equipment, tools and utensils used should be clean. Personnel engaged in herbal processing should maintain hygiene and wear protective clothing, gloves, head/hair covering and footwear [19].

TMPs reported lack of knowledge on preservation techniques and low earnings from selling nutraceutical plants. During storage, nutraceutical plants should be kept in a clean hygienic place to avoid contamination and formation of aflatoxins [19]. Nutraceutical plants should be protected from microbial insect, rodent and other pest contamination. Packaging should provide the best protection against moisture, light, heat and physical damage to the processed nutraceutical materials [19]. Stores should be clean, well-lit and have acceptable temperature [19]. Most of the TMPs did not keep records on infections treated or nutraceutical plants used. This was also reported by Addo-Fordjour [110], where no records of treatment were written down by TMPs, as they mostly depend on memory. According to WHO [19] record keeping is important in drug administration. Mahomoodally [111] reported that proper validation of traditional knowledge and quality control standards are lacking in Africa and these are some of the limitations to the growth of a modern African nutraceutical industry, compared to Europe and Asia, where Traditional methods and formulations have been recorded and evaluated both at national and local levels [111].

## Conclusion

This study demonstrates the availability and diversity of nutraceutical plants in Northern Uganda and reports traditional methods used by TMPs during processing and administration. Both men and women used nutraceutical plants to manage opportunistic infections associated with HIV/AIDS and showed great extent of their traditional knowledge. Age and gender are the main factors that seem to influence the ethnobotanical knowledge of respondents. The observed Traditional methods and formulations used by TMPs do not guarantee safety and quality assurance due to several problems reported. Most of the nutraceutical plants in the study area are wild and abundant; however, high percentage use of plant roots and bark threatens the sustainable use from the wild.

## Recommendations

### Recommendations to National Forestry Authority, Uganda

Harvesting of nutraceutical plants from forests in Northern Uganda should be regulated by National Forestry Authority in Uganda. This will contribute to efforts to preserve and promote Nutraceutical plants in Northern Uganda.

## Recommendations to National drug Authority, Uganda

The National Drug Authority should expand their operations to other regions of Uganda and set up regulatory control measures and standards to be used during collection, processing and storage. Quality assurance during production and utilisation of nutraceutical plants needs to be taken into account, since it directly impacts the safety and efficacy of nutraceutical plant products. Ministry of Education in Uganda is called upon to convene and train TMPs from all regions in Uganda about basic knowledge on diagnosis of disease, record maintenance, Legal systems, business management, processing and packaging nutraceutical plants for commercial use, how to maximize profits and protect their knowledge.

### Recommendation to the Ministry of Health Uganda

Due to this high degree of medical pluralism, collaboration and communication between biomedical clinicians and Traditional medicine practitioners should be encouraged.

### Recommendation to Gulu University and Registrar of Patents office, Uganda

Since Contemporary Intellectual Property Law permits only the Patenting of an identified Active Principle from a plant, not the plant or folk information relating to Medicinal properties of a plant [112], Copyright and Intellectual Property Rights (IPR) issues of Traditional medicine practitioners can be fully exploited with the help of Gulu University and Registrar of Patents office, Uganda.

## Data Availability

The data sets during and /or analysed during the current study are available from the corresponding author on reasonable request.

## Appendix

See Tables 8, 9.

**Table 8:** Nutraceutical plants in Gulu, Pader and Amuru districts used to manage bacterial opportunistic infections and mode of preparation for quality control

Scientific name, Family, Local name in native ‘Acholi dialect’	Accession number	Habit	Habitat	Conservation status	Bacterial opportunistic infection treated (uses)	Part used	Mode of preparation and administratn	Familiarity index	Fidelity Level
*Acacia hockii* De Wild *(*Leguminoceae) Okuto-oriang	MHU 32118	shrub	Bu	W/A	Cough. Diarrhea, stomachache	FL	Mix leaves in hot water, drink infusion	0.058	0.115
*Acalypha villicaulis* Hochst (Euphorbiaceae). Ayila	MHU 34107	Shrub	Bu	W/R	Diarrhea, eye infection	FR	Boil roots, drink decoction	0.028	0.110
*Afromomum angustifolium* Sonn (Zingiberaceae) Oceyo	MHU 31591	Herb	Bu	W/R	diarrhoea	FL	Boil leaves, drink decoction	0.003	0.001
*Albizia coriaria* Welw.ex.Oliv (Leguminoceae Ayek–ayek	MHU 38528	Tree	Wo	W/R	Tuberculosis, chest infection, sore throat	FR	Boil roots, drink decoction	0.023	0.110
*Albizia grandibracteata* Taub Leguminoseae Awak Owak	MHU 34331	Tree	Wo	W/A	Eye infection	FL	Macerate leaves in cold water, drink extract	0.003	0.010
*Ammocharis tinneana* (Kotschy & Peyr.) Amaryllidaceae Joda	MHU 41025	Bulb	Bu	W/R	cough	FL	Soak leaves in cold water, drink maceration	0.003	0.01
*Ampelocissus africana* (Lour.)Merr Vitaceae Olok	MHU 41684		Shrub	W/R	Diarhhoea	FR	Boil roots, drink decoction	0.008	0.03
*Annona muricata* L Annonaceae Obvolo	MHU 51003	Shrub	Bu	W/A	Diarrhea. stomachache, chest infection	FR	Boil roots, drink decoction	0.025	0.05
*Asparagus africana* Lam Asparagaceae Lagwari	MHU 35783	Herb	Fo	W/R	Eye infection	FFr	Swallow fruits	0.005	0.02
*Azadirachta indica* A.Juss Meliaceae Neem (English)	MHU 50969	Tree	P	Cu/A	Cough, diarrhea, tb, stomachache	FL	Boil leaves in water, drink decoction	0.025	0.02
*Carica papaya.*L Caricaceae Papai	MHU 42266	Herb	Ho	Cu/A	Diarrhea, sore throat, stomachache	FR	Boil roots, drink decoction	0.03	0.06
*Carisa edulis* (Forssk) Vahl Apocynaceae Ocuga	MHU 35814	Spiny shrub	Fo	W/A	Sore throat, cough	FL	Mix leaves in hot water, drink infusion	0.005	0.005
*Cassia singueana* Delile Leguminoseae Lakodok /akwadok	MHU 39624	Tree	Bu	W/R	Eye infection	S	Squeeze sap in eye	0.003	0.01
*Cassia nigricans* Vahl Leguminoseae Abanceng	MHU 32957	Shrub	Bu	W/A	Abdominal pain, cough, sore throat	FL	Mix leaves with hot water, drink infusion	0.045	0.09
*Citrus limon*(L.)Burm Rutaceae Lemun	MHU 42222	Shrub	Ho	Cu/A	Cough	FFr	Squeeze fruit in water, drink	0.008	0.003
*Clematis hirsuta* (Perr. & Guill.) Ranunculaceae Omwombyer(Acholi)	MHU 32013	Shrub	Fo	W/R	Cough, sore throat, stomachache, diarrhoea	FR	Boil roots, drink decoction	0.008	0.030
*Clerodendrum myricoides* (Hochst.)R.Br.ex Vatke Lamiaceae Okwero (Acholi)	MHU 38184	Shrub	Fo	W/R	Cough, eye infection, stomachache	FR	Boil roots in water, drink decoction	0.018	0.070
*Echinops amplexicaulis* Oliv Astereceae Alilokwang	MHU 37710	Shrub	Bu	W/R	Ear infection	FR	Boil roots, drink decoction	0.003	0.01
*Erigeron floribundus* (kunth) Asteraceae Aditong/ Gwelworu	MHU 51148	Herb	Rs	W/A	Cough, sore throat	FL	Chew leaves, infusion leaves	0.028	0.004
*Erythrina abyssinica* Lam.ex DC Leguminoceae Lucoro	MHU 42157	Tree	Fo	W/A	Eye and ear infection, diarrhea, wounds	FSB	Boil bark, drink decoction	0.013	0.013
*Euphorbia hirta* L Euphorbiaceae Acak–acak	MHU 42795	Herb	Rs	W/A	Syphilis, wounds stomachache	S	Apply sap on warts	0.01	0.02
*Euphorbia tirucalli* L Euphorbiaceae Kilajok	MHU 50984	Shrub	Bu	W/A	Eye infection, cough, eye infection, diarrhoea	S	Squeeze sap into eye	0.063	0.250
*Ficus sycomorus* L Moraceae Olam	MHU 37779	Tree	Bu	W/A	Syphilis, diarrhea, stomachache	FL,FSB	Boil leaves or bark in water, drink decoction	0.048	0.011
*Ficus vasta* Forssk Moraceae Foyo	MHU 36129	Tree	Bu	W/R	Diarrhea	FL	Mix leaves in hot water, drink infusion	0.003	0.01
*Gardenia ternifolia* Schum.& Thorn Rubiaceae Odwong	MHU 35775	Tree	Bu	W/A	Ear infection, tb, cough	S	Roast leaves, drip sap in ear	0.013	0.025
*Gynandropsis gynandra* (L.) Cleomeaceae Akeyo	MHU 35828	Herb	Ga	Cu/A	Ear and eye infection	FR	Boil roots, drink decoction	0.043	0.015
*Harisonia abyssinica* Oliv Rutaceae Pedo	MHU 35837	Shrub	Bu	W/R	Tuberculosis, cough, eye infection, diarrhoea	FL	Mix leaves in hot water, drink infusion	0.020	0.03
*Harungana madagascariensis* Lam. Ex Poiret Hypericaceae Aremu	MHU 32681	Big shrub	Fo	W/R	Diarrhea, stomachache	FR	Boil roots, drink decoction	0.023	0.09
*Hibiscus sabdariffa* (L.) Malvaceae Malakwang	MHU 41711	Shrub	Ga	Cu/A	Eye infection	FL	Cook leaves, eat as vegetable	0.005	0.010
*Hoslundia opposita* Vahl Lamiaceae Tuutu	MHU 41663	Shrub	Bu	W/R	Diarrhea, sore throat	FR	Boil roots, drink decoction	0.008	0.006
*Hyparrhenia rufa* (Nees) Stapf Poaceae Ajuu	MHU 33697	Grass	Gr	W/A	Eye infection	FR	Boil roots, drink decoction	0.003	0.01
*Indigofera arrecta* Hochst. A.Rich Leguminoceae Laywer	MHU 33709	Shrub	Bu	W/A	Stomachache. Cough, sore throat, tb	FR	Boil roots, drink decoction	0.030	0.017
*Khaya senegalensis* (Desr.) A.Juss Meliaceae Tido	MHU 42488	Tree	Bu	W/A	Diarrhoea, cough, stomachache	FSB	Boil bark, drink decoction	0.028	0.005
*Kigelia africana* (Lam.)Benth Bigoniaceae Yago	MHU 31702	Tree	Fo	W/R	Cough	FR	Boil roots, drink decoction	0.005	0.02
*Lantana camara* L Verbenaceae Abelwinyu	MHU 50924	Shrub	Bu	W/A	Sore throat, ear infection, cough, eye infection	FL	Mix leaves with hot water, drink infusion	0.010	0.02
*Lippia javanica* (Burm.f.) Spreng Verbenaceae Orwo	MHU 31966	Herb	Bu	W/R	Sore throat, eye infection	FL	Chew leaves	0.003	0.03
*Lonchocarpus laxiflorus* Guill &Perr Leguminoceae Olwedo	MHU 43280	Tree	Fo	W/A	T.B, Respiratory tract infection, chest pain	FL	Mix leaves in hot water, drink infusion	0.020	0.011
*Moringa oleifera* L Moringaceae Moringa	MHU 41303	Tree	Wo	Cu/A	Stomachache, cough	FL	Mix leaves in hot water, drink infusion	0.008	0.010
*Musa paradisiaca* L Musaseae Labolo	AAA-EA	Herb	P	Cu/A	Diarrhea, wounds	S	Mix sap in cold water, drink	0.018	0.009
*Occimum gratissimum* L Lamiaceae Mida	MHU 35677	Herb	Rs	W/A	Cough	FL	Mix leaves in hot water, drink infusion	0.005	0.001
*Persea americana* Mill Anacardiaceae Avocado (English)	MHU 42243	Tree	Ho	Cu/A	Chest infection	FL	Boil leaves, drink decoction	0.003	0.001
*Physalis minima* Linn Solanaceae Kong-ogwal	MHU 37418	Herb	Rs	W/A	Sore throat	FR	Boil roots, drink decoction	0.003	0.010
*Pistia stratiotes* L Araceae Lagada	MHU 40124	Herb	Sw	W/R	Eye infection, cough	S	Drip leaf sap in eye	0.010	0.04
*Pseudocedrella kotschyi* (Schweinf.) Meliaceae Ofuti	MHU 50947	Tree	Bu	W/A	Diarrhoea	FR	Boil roots, drink decoction	0.008	0.003
*Psidium guajava* L Myrtaceae ‘ Mupeera	MHU 42105	Tree	Ho	Cu/α	Cough	FL	Boil leaves, drink decoction	0.010	0.04
*Sansevieria newtoniana T.G* Asparagaceae Tworo-gwok	MHU 42552	Herb	Bu	W/R	Diarrhea, ear infection	FL	Macerate leaves by soaking in cold water, drink extract	0.010	0.004
*Sarcocephalus latifolius* Smith Rubiaceae Munyu	MHU 50529	Tree	Bu	W/A	Diarrhea, syphillis	FR,FSB	Boil roots or bark in water, drink decoction	0.008	0.004
*Senna obtisifolia* (L.) Leguminoseae Oyedo	MHU 36690	Shrub	Fo	W/R	Eye infection, sore throat	S	Squeeze sap in eye	0.005	0.010
*Solanecio manii* (Hook.f.)C Solanaceae Taa-lyec	MHU 50551	Shrub	Bu	W/A	Abdominal pain, chest pain	FR	Boil roots in water, drink decoction	0.008	0.003
*Solanum aethiopicum* L Solanaceae Tula	MHU 32755	Shrub	Ga	Cu/A	Diarrhea	FFr	Cook fruits, eat as vegetable	0.003	0.01
*Sporobolus pyramidalis* P.Beauv Poaceae Ajiki (Acholi)	MHU 40673	Grass	Gr	W/A	Cough, sore throat	FL	Mix leaves in hot water, drink infusion	0.010	0.04
*Steganotaenia araliacea* Hochst Apiaceae Olwiro	MHU 38029	Shrub	Fo	W/R	Syphilis, T.B, respiratory tract infection	FR	Boil roots in water, drink decoction	0.018	0.035
*Tamarindus indica* L Leguminoseae Cwaa	MHU 40035	Tree	Ho	Cu/A	Diarrhea	FL,FSB	Boil leaves or root, drink decoction	0.003	0.010
*Teclea nobilis Delile* Rutaceae Acha–acha	MHU 32391	shrub	Bu	W/R	Stomachache	FL,FR	Mix leaves, roots in hot water, drink infusion	0.015	0.06
*Terminalia glaucescens* Planch.ex.Benth Combretaceae Opok	MHU 42410	Tree	Fo	W/R	Cough, stomachache, syphilis	FL	Chew leaves, boil roots in water, drink decoction	0.015	0.06
*Uapaca guineensis* Mull-Arg Euphorbiaceae Acak	MHU 32266	Tree	Bu	W/A	Syphilis, stomachache	FR	Boil roots, drink decoction	0.003	0.020
*Vernonia amygdalina* Del Asteraceae Labwori	MHU 42532	Shrub	Bu	W/A	Cough, diarrhea, stomachache	FL	Mix leaves in hot water, drink infusion	0.030	0.003
*Vernonia lasiopus* O.Hoffm Asteraceace Labuka	MHU 35754	Shrub	Bu	W/R	Eye and ear infection, stomachache	S	Squeeze sap into ear, eye	0.048	0.095
*Vigna unguiculata* (L) Walp Leguminoceae Boo	MHU 35800	Herb	Ga	Cu/A	Ear infection	FL	Cook leaves, eat as vegetable	0.030	0.01
*Vitellaria paradoxa* Gaetn Sapotaceae Yaa	MHU 33728	Tree	Ho	Cu/A	Diarrhea, stomachache	FSe	soak crushed seeds in cold water by maceration, drink	0.018	0.01
*Vitex doniana* L Verbenaceae Oywelo	MHU 35770	Tree	Bu	W/A	Diarrhea, chest pain, tb, ear and eye infection	FR	Boil roots in water, drink decoction	0.015	0.060
*Waburgia ugandensis* Sprague Canellaceae Abaki	MHU 50888	Tree	Bu	W/R	Tuberculosis, respiratory tract infection, chest pain, stomachache	FSB	Boil bark in water, drink decoction	0.020	0.080
*Zingziber officinale* (L)H.Karst Zingiberaceae Tangawuzi	MHU 51051	herb	Ga	Cu/A	Cough	FR	Boil roots drink decoction	0.008	0.030
*Zizyphus abyssinica* Hochst Rhamnaceae Lango	MHU 33483	Shrub	Bu	W/A	Ear infection, diarrhea	FL	Mix leaves in cold water, drink extract	0.005	0.020

Parts used: FL: fresh leaf, FR: fresh Root, FSB: fresh stem bark, FSe: fresh seed, FFr: fresh fruit, Bu: bulb, S: sap

Habitat: Bu—bushland, Gr—grassland, Ho—homestead, Rs—roadside, P—plantation, Sw—swamp, Ga—garden, Fo—forest, Wo—woodland

Conservation status: W/A: wild and abundant, W/R: wild and rare, Cu/A: cultivated and abundant, Cu/R: cultivated and rare

Decoction: water extraction by boiling of dissolved plant material and drank

Infusion: plant material is added to hot water and left to stand for few minutes and drank

**Table 9:** Nutraceutical plants used to manage fungal opportunistic infections and mode of preparation for quality control

Scientific name, Family, Local name in native ‘Acholi dialect’	Accession number	Habit	Habitat	Conservation status	Fungal opportunistic infection (uses)	Part used	Mode of preparation	Familiarity Index	Fidelity level
*Anacardium occidentale* L Anacardiaceae Fulmunnu	MHU 42396	T	Ho	Cu/A	Oral candidiasis	FB	Boil bark, drink decoction	0.015	0.020
*Artocarpus heterophyllus* Lam Moraceae Fene	MHU 40229	T	Ho	Cu/A	Oral candidiasis	FR	Boil roots, drink decoction	0.003	0.005
*Cajanus cajan *(L.) Millsp*.* Leguminoceae Lapena	MHU 51151	S	Ga	Cu/A	Oral candidiasis	FL	Chew leaves	0.003	0.0001
*Carisa edulis* (Vahl.) Apocynaceae Ocuga	MHU 35814	S		W/A	Oral candidiasis	FL	Boil leaves, drink decoction	0.005	0.0100
*Citrus limon* (L.) Burm Rutaceae Lemun	MHU 42222	S	Ho	Cu/A	Skin fungal infection	FFr	Rub fruit peel on skin	0.003	0.0009
*Clerodendrum swienfuthii* L Lamiaceae Lacer	MHU 40967	S	Bu	W/R	Oral candidiasis	FL	Boil leaves, drink decoction	0.015	0.0030
*Cucurbita pepo* L Cucurbitaceae Okono	MHU 42492	C	Ga	Cu/A	Skin fungal infection	FL	Rub leaves on skin	0.003	0.0007
*Cucurma longa* L Zingiberaceae Binzaali	MHU OJ55	H	Ga	Cu/A	Oral candidiasis	DR	Mix powdered root in water, drink	0.003	0.010
*Erythrina abyssinica* Lam Leguminoseae Lucoro	MHU 32166	T	Bu	W/A	Oral candidiasis	FR	Boil roots, drink decoction	0.010	0.002
*Euphorbia hirta* L Euphorbaiceae Acak–acak	MHU 50984	H	Bu	W/A	Skin fungal infection, oral candidiasis	FL	Rub leaves on skin	0.005	0.001
*Harisonia abyssinica* Oliv Rutaceae Pedo	MHU 35837	S	Bu	W/R	oral candidiasis	FL	Boil leaves, drink decoction	0.005	0.005
*Harungana madagascariensis* Lam. Ex Poir Hypericaceae Aremu	MHU 32681	S	Bu	W/R	Skin fungal infection	FL	Boil leaves, drink decoction	0.003	0.001
*Hoslundia opposita* Vahl Lamiaceae Tuutu	MHU 31950	S	Bu	W/R	Oral candidiasis. Candida, skin fungal infection	FR	Boil roots, drink decoction	0.010	0..008
*Imperata cylindrica* (L.) Poaceae Obiya	MHU 35056	G	Gr	W/A	Oral candidiasis	FR	Boil roots, drink decoction	0.003	0.0014
*Indigofera arrecta* Hochst.ex A.Rich Leguminosae Laywer	MHU 42857	S	Bu	W/A	Oral candidiasis	FL	Boil leaves, drink decoction	0.003	0.0014
*Lantana camara* L Verbenaceae Abelwinyu	MHU 50924	S	Bu	W/A	Oral candidiasis	FL	Soak leaves in hot water, drink infusion	0.005	0.005
*Lippia javanica* Burm.f. spreng Verbenaceae Orwo	MHU 31966	S	Bu	W/R	Skin fungal infection	FL	Boil leaves, drink decoction	0.003	0.003
*Lonchocarpus laxiflorus* Guill. & Perr Leguminoseae Olwedo	MHU 43280	T	Bu	W/R	Oral candidiasis, Skin fungal infection	FR	Boil roots, drink decoction	0.010	0.0036
*Luffa cylindrical* (Linn.) M.J.Roem Cucurbitaceae Amoo	MHU 41714	C	Ga	Cu/A	Skin fungal infection	FL	Rub leaves on skin	0.013	0.005
*Moringa oleifera* Lam Moringaceae Moringa (scientific)	MHU 50971	T	Ho	Cu/A	Oral candidiasis	FL	Boil leaves, drink decoction	0.005	0.003
*Musa paradisiaca* L Musaceae Labolo	AAA-EA	H	p	Cu/A	Oral candidiasis, skin fungal infection	S	Mix sap in water, drink	0.010	0.0067
*Piliostigma thonningii* (Schumach) Milne-Redh Leguminoseae Ogali	MHU 35791	S	Bu	W/A	skin fungal infect, oral candidiasis	FL	Boil leaves, drink decoction	0.010	0.0014
*Psidium guajava* L Myrtaceae Mupera	MHU 42105	T	Ho	Cu/A	Oral candidiasis	FL	Soak leaves in hot water, drink infusion	0.003	0.002
*Ricinus communis* L Euphorbaceae Coga macon	MHU 42123	S	Bu	W/A	Oral candidiasis	FL	Boil leaves, drink decoction	0.003	0.005
*Securidaca longipenduculata* Fresen polygalaceae lalia	MHU 35766	T	Bu	W/R	Skin fungal infection	FR	Boil roots, drink decoction	0.003	0.0025
*Senna obtisifolia* L Leguminoceae oyedo	MHU 36690	S	Bu	W/A	Skin fungal infection	FR	Boil leaves, drink decoction	0.003	0.0033
*Solanum incanum* L Solanaceae Ocok	MHU 35802	S	Rs	W/A	Oral candidiasis	FL	Boil roots, drink decoction	0.003	0.0003
*Vernonia amygdalina* Del Astereceae Labwori	MHU 42532	S	Bu	W/A	Skin fungal infection, oral candidiasis	FL	Boil leaves, drink decoction	0.018	0.0015
*Vernonia lasiopus O. Hffm* Asteraceae Labuka	MHU 35754	S	Bu	W/R	Candida of genitals	FL	Rub leaves on gentitals	0.003	0.0005
*Vitellaria paradoxa* C.F.Gaetn Verbenaceae Yaa	MHU 33728	T	Ho	Cu/A	Oral candidiasis	FSe	Soak crushed seeds in cold water by maceration, drink	0.015	0.0088

Parts used: FL: fresh leaf, FR: fresh Root, FSB: fresh stem bark, FSe: fresh seed, FFr: fresh fruit, Bu: bulb, S: sap

Habitat: Bu—bushland, Gr—grassland, Ho—homestead, Rs—roadside, P—plantation, Sw—swamp, Ga—garden, Fo—forest, Wo—woodland

Habit: S—shrub, T—tree, H—herb, C—climber, G—grass

Conservation status: W/A: wild and abundant, W/R: wild and rare, Cu/A: cultivated and abundant, Cu/R: cultivated and rare

Decoction: water extraction by boiling of dissolved plant material and drank

Infusion: plant material is added to hot water and left to stand for few minutes and drank

## Abbreviations

A.R.T: Anti-retroviral therapy
ARV: Antiretroviral drugs
C.D.C: Centres for disease control and prevention
F. I: Familiarity index
F.L.I: Fidelity level
HIV/AIDS: Human immunodeficiency virus/acquired immunodeficiency syndrome
I.C.F: Informant consensus factor
S.T.D: Sexually transmitted diseases
T.M.P: Traditional medicine practitioner
UNAIDS: United Nations Programme on HIV/AIDS
W.H.O: World Health Organization
PLHIV: People living with HIV
